# Enamel matrix derivative and root coverage: A literature review

**DOI:** 10.21142/2523-2754-1302-2025-244

**Published:** 2025-05-16

**Authors:** Edgar Daniel Vargas Quiroga, Brenda Carolina Pattigno Forero, Gloria Estefanía Amaya Chica, Lesbia Rosa Tirado Amador, Marilia Bianchini Lemos Reis

**Affiliations:** 1 Department of Oral & Maxillofacial Surgery and Periodontology, Ribeirao Preto Dental School (FORP/USP), University of Sao Paulo. Ribeirao Preto, Sao Paulo. Brazil. edgardanielvargasquiroga@gmail.com, g.amayachica@gmail.com, marilia.bianchini.reis@gmail.com Universidade de São Paulo Department of Oral & Maxillofacial Surgery and Periodontology Ribeirao Preto Dental School (FORP/USP) University of Sao Paulo Ribeirao Preto Sao Paulo Brazil edgardanielvargasquiroga@gmail.com g.amayachica@gmail.com marilia.bianchini.reis@gmail.com; 2 Department of Pediatric Dentistry, Riberao Preto Dental School (FORP/USP), University of Sao Paulo. Ribeirao Preto, Sao Paulo, Brazil. brendacaropf@gmail.com Universidade de São Paulo Department of Pediatric Dentistry Riberao Preto Dental School (FORP/USP) University of Sao Paulo Ribeirao Preto Sao Paulo Brazil brendacaropf@gmail.com; 3 Professor and researcher, Group GINOUS, University of Sinu Cartagena. Cartagena, Colombia, lesbia.tirado@gmail.com Group GINOUS University of Sinu Cartagena Cartagena Colombia lesbia.tirado@gmail.com

**Keywords:** gingival recession, root coverage, enamel matrix derivative, mucogingival surgery, recesión gingival, recubrimiento radicular, matriz derivada del esmalte, cirugía mucogingival

## Abstract

**Introduction:**

: The use of enamel matrix derivative proteins, also known as Emdogain®, has revolutionized clinical practice in dentistry since the 90s. This biomaterial has demonstrated to promote periodontal tissues regeneration lost due to periodontal disease which persists as a public health problem, emerging as an effective alternative in treatments such as non-surgical periodontal therapy, treatment of furcation lesions, intraosseous defects, and more recently, its use has become especially popular in root coverage surgeries.

**Objective:**

: Evaluate studies in reference to the use of enamel-derived matrix proteins in periodontal treatments, to offer a comprehensive and up-to-date vision of its impact and effectiveness.

**Materials and Methods:**

: Systematic review was conducted about randomized clinical trials (RCTs) published between 2019 and 2024, using databases PubMed and Scopus, considering Clinical measurements performed in the studies.

**Results:**

The analyzed studies compared different surgical techniques for the treatment of gingival recessions, focusing on the percentage of root coverage (RC), clinical attachment gain (CAL), increase in gingival thickness (KTW), long-term stability, and postoperative pain (VAS).

**Conclusion:**

EMD significantly contributes to improvement of periodontal regeneration and long-term outcomes of root coverage surgeries, results obtained show a lot of variability. Randomized controlled trials are necessary to evaluate efficacy in different clinical contexts.

## RESUMEN

**Introducción:**

: El uso de las proteínas derivadas de la matriz del esmalte, conocidas como Emdogain®, ha revolucionado la práctica clínica en odontología desde los años 90. Este biomaterial promueve la regeneración de tejidos periodontales perdidos por enfermedad periodontal (problema de salud pública) y emerge como alternativa efectiva en terapia periodontal no quirúrgica, tratamiento de lesiones de furcación, defectos intraóseos y, más recientemente, en cirugías de cobertura radicular.

**Objetivo:**

Evaluar estudios referentes al uso de proteínas de matriz derivadas del esmalte en tratamientos periodontales, para ofrecer una visión integral y actualizada de su impacto y efectividad.

**Materiales y métodos:**

Se realizó una revisión sistemática de ensayos clínicos aleatorizados (ECA) publicados entre 2019 y 2024, utilizando las bases PubMed y Scopus, considerando mediciones clínicas de los estudios.

**Resultados:**

Los estudios compararon diferentes técnicas quirúrgicas para el tratamiento de las recesiones gingivales, centrándose en el porcentaje de cobertura radicular, ganancia de inserción clínica, aumento del grosor gingival, estabilidad a largo plazo y dolor posoperatorio.

**Conclusión:**

La EMD contribuye significativamente a mejorar la regeneración periodontal y los resultados a largo plazo de las cirugías de cobertura radicular. Los hallazgos son variables y son necesarios ensayos clínicos controlados y aleatorizados para evaluar la eficacia en diferentes contextos clínicos.

## INTRODUCTION

One of the current concerns among patients regarding aesthetics and dental sensitivity is gingival recession, which is an apical displacement of the gingival margin relative to the cemento-enamel junction (CEJ), exposing the root surface to the oral environment ^1^^,^^2^. Its etiology includes traumatic factors such as aggressive tooth brushing, excessive occlusal forces, and trauma, in combination with the patient's anatomical and biological predisposing factors, such as a thin gingival phenotype, dental malpositions, thin alveolar bone, and dental malformations ^3^^,^^4^. Periodontal mucogingival surgery aims to restore lost anatomy through surgical techniques designed to reposition the gingival margin either coronally or at the level of the cemento-enamel junction (CEJ) ^5^. The use of subepithelial connective tissue grafts (SCTG) has been shown to be effective in achieving increases in tissue volume and quality; however, it often results in the formation of a long junctional epithelium ^6^^,^^7^.

In 1997, Lars Hammarström introduced enamel matrix derivatives (EMD), which are porcine-derived proteins, with amelogenins as the main component ^8^. This biomaterial, commercially known as Emdogain®, has become a valuable tool in procedures such as non-surgical periodontal therapy, furcation defect treatment, and intrabony defect regeneration. Its use has gained popularity in root coverage surgeries ^9^^-^^11^. These proteins mimic the biological processes that occur during tooth development, promoting the formation of new acellular extrinsic fiber cementum, periodontal ligament, and alveolar bone ^12^. EMD stimulates cell proliferation and differentiation, particularly of fibroblasts and cementoblasts, which are responsible for extracellular matrix release. Additionally, it contributes to an increase in vascular endothelial growth factor (VEGF), enhancing tissue oxygenation and inducing angiogenesis. Moreover, it promotes transforming growth factor-beta (TGF-β), facilitating tissue repair and supporting the expression of gingival fibroblasts and periodontal ligament cells ^12^^,^^13^. The regenerative effect of EMD on bone is limited ^14^. While it has the potential to induce bone formation, it appears to be insufficient on its own and should be supplemented with a bone graft for optimal results ^14^^,^^15^.

Its association with periodontal root coverage surgery has shown improvements in connective tissue attachment, primarily because it promotes the formation of cementum and periodontal ligament. However, its effect on bone formation is limited. Nevertheless, it enhances the insertion of connective tissue fibers into the root surface ^15^. The objective of this narrative review is to thoroughly analyze contemporary studies from the last five years on randomized clinical trials investigating the use of enamel matrix derivative (EMD) in root coverage procedures, providing a comprehensive and updated perspective on its impact and efficacy.

## MATERIAL AND METHODS

The search and analysis of information followed a systematic approach to identify, select, and analyze relevant studies published in the last five years. 

### Search Strategy

A search was conducted in two databases: PubMed and Scopus, with the aim of identifying research published between 2019 and 2024. The search method used a combination of keywords and Boolean operators to ensure a comprehensive selection of articles:

### PubMed:

*(("enamel matrix derivative"[All Fields] OR "EMD"[All Fields]) AND ("root coverage"[All Fields] OR "gingival recession"[All Fields]) AND ("surgery"[All Fields] OR "surgical procedure"[All Fields]) AND ("randomized controlled trial"[All Fields] OR "RCT"[All Fields])) AND (y_5[Filter])*

### Scopus:

*TITLE-ABS-KEY ((“enamel matrix derivative" OR emd ) AND ( "root coverage" OR "gingival recession" ) AND (“surgery" OR "surgical procedure" ) AND ( "randomized controlled trial" OR rct ) ) AND PUBYEAR > 2018 AND PUBYEAR < 2025*

### Embase:

*#1 ('enamel matrix derivative'/exp OR 'enamel matrix derivative' OR 'emd'/exp OR 'emd') AND ('root coverage'/exp OR 'root coverage' OR 'gingival recession'/exp OR 'gingival recession') AND ('surgery'/exp OR 'surgery' OR 'surgical procedure'/exp OR 'surgical procedure') AND ('randomized controlled trial'/exp OR 'randomized controlled trial' OR 'rct')*

*#2 (2019:py OR 2020:py OR 2021:py OR 2022:py OR 2023:py OR 2024:py)*

*1# AND 2#*

The search was limited to randomized clinical trials (RCTs) with a follow-up period of more than 6 months, published in English within the last five years.

### Inclusion and Exclusion Criteria

The following were excluded:

1. Articles that were not RCTs (e.g., case reports, observational studies, systematic reviews, or scoping reviews).
2. Articles that did not focus on root coverage surgery or EMD (e.g., surgeries for intraosseous defects).
3. Articles not published in English.
4. Articles published outside the specified five-year period.

### Study Selection Process

The study selection process was carried out in three stages:

1. Screening: Titles and abstracts of all retrieved studies were reviewed to exclude irrelevant articles.
2. Review: The remaining articles were evaluated in full to determine their eligibility based on the inclusion and exclusion criteria.
3. Selection: Studies that met all the criteria were included in the review. Additionally, the Jadad scale was used to assess the methodological quality of the clinical trials.

The Rayyan tool was used to detect and eliminate duplicate articles. Two independent reviewers, B.C.P.F. and E.D.V.Q., conducted the selection process. Disagreements between reviewers were resolved by a third reviewer, G.E.A.C., who determined whether the article would be included or excluded. The search was limited to studies published between 2019 and 2024. 

### Data Extraction

The following data were extracted from the selected studies: Author/Year, Country, Sample Size, Surgical Technique, Emdogain Group, Control Group, Outcomes (Root Coverage Percentage and Clinical Attachment Gain), Key Findings, and Conclusions. The Jadad Scale ^16^^,^^17^ was used to evaluate the methodology and assign a score to each selected clinical trial. The details of the selection process are presented in Table 1. The data were organized in an Excel table to facilitate collection, organization, and analysis of the information.

**Table 1:** Criteria used to assess the methodological quality of the included studies

Criterion	Description
Author	Name
Year	Year of publication of the study.
Impact Factor	Web of Science (WoS) Journal Info
Quartile (Q1-Q4)	Journal ranking within the Web of Science (WoS) Journal Info, ranging from Q1 (highest) to Q4 (lowest).
Randomization	Indicates whether the study was randomized (Yes/No).
Randomization Method	Assesses whether the randomization was adequate, inadequate, or not described.
Double-Blind	Indicates whether the study used the double-blind technique (Yes/No).
Blinding Method	Assesses whether blinding was adequate, inadequate, or not described.
Reported Dropouts/Withdrawals	Indicates whether the study reported participant losses and withdrawals during follow-up (Yes/No).
Jadad Score (0-5)	Jadad score for assessing the methodological quality of randomized clinical trials, considering randomization, blinding, and reporting of losses and exclusions.

## RESULTS

**Figure 1:**
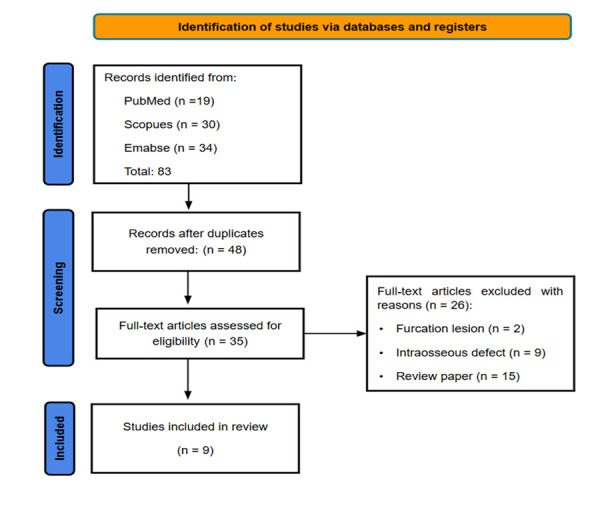
Flow-chart of the screening of the relevant publications

A total of 83 studies were identified in the PubMed, Scopus, and Embase databases. After removing duplicates and applying the selection criteria filters, 9 studies were included in this review Figure 1 and Table 2.

**Table 2:** Selected articles and methodological quality assessment based on the Jadad scale

Article	Year	Impact Factor	Quartile (Q1-Q4)	Randomized? (Yes/No)	Randomization Method (Adequate/Inadequate/Not described)	Double-Blind? (Yes/No)	Blinding Method (Adequate/ Inadequate/Not described)	Reported Dropouts/ Withdrawals? (Yes/No)	Escore Jadad (0-5)
Górski *et al*. (^18^)	2020	3.1	Q1	Yes	Adequate	Yes	Adequate	Yes	5
França-Grohmann *et al*. (^19^)	2019	3.1	Q1	Yes	Adequate	Yes	Adequate	Yes	5
Mercado *et al*. (^20^)	2019	4.2	Q1	Yes	Adequate	Yes	Adequate	Yes	5
Stähli *et al*. (^21^)	2020	3.1	Q1	Yes	Not described	No	Adequate	Yes	3
Zuhr *et al*. (^22^)	2020	5.8	Q1	Yes	Not described	No	Adequate	Yes	3
Dias *et al*. (^23^)	2022	3.1	Q1	Yes	Not described	No	Adequate	Yes	3
Stähli *et al*. (^24^)	2023	3.1	Q1	Yes	Adequate	No	Adequate	Yes	4
Rieder *et al*. (^25^)	2024	3.1	Q2	Yes	Adequate	No	Not described	Yes	3
Aydinyurt *et al*. (^26^)	2019	1.5	Q3	Yes	Inadequate	Yes	Adequate	Yes	3

**Table 3:** Summary of the included articles. Abbreviation: RCT- randomised controlled trial. MCAT - Modified Coronally Advanced Tunnel. SCPF - Semilunar Coronally Positioned Flap. CAF - Coronally Advanced Flap. TUN - Tunnel Technique. SCTG - Subepithelial Connective Tissue Graft. CTG - Connective Tissue Graft. XDM - Xenogeneic Dermal Matrix. CRC - Complete root coverage.

Author/year	Study Design	Sample Size (patients)	Gingival recession	Surgical Technique	Emdogain Group	Group Control	Key Findings
Górski *et al*. 2020 (^18^)	RCT	20	RT 1 and RT 2 Cairo	MCAT	MCAT + SCTG + EMD	MCAT + STCG	No significant difference in root coverage between the two groups (87.4% for test vs. 90.9% for control). Complete root coverage (CRC) was similar in both groups (86.7% for test vs. 85.3% for control).
França-Grohmann *et al*. 2019 (^19^)	RCT	30	Miller Class I (RT 1 Cairo)	SCPF	SCPF + EMD	SCPF alone	No significant difference in root coverage between the two groups (90.86% for test vs. 79.76% for control). CRC was higher in the test group (66.67% vs. 33.33% in control).
Mercado *et al*. 2019 (^20^)	RCT	41	Multiple Miller Class III and IV (RT 2 and 3 Cairo)	CAF	CTG + EMD	CTG alone	In the test group, recession depth decreased from 5.71 ± 0.58 mm to 1.57 ± 0.85 mm at 36 months, while in the control group, it decreased from 5.94 ± 0.46 mm to 2.51 ± 0.62 mm. The difference was statistically significant ( P<0.001). In the test group, KT increased from 1.51 ± 0.26 mm to 4.18 ± 0.34 mm, while in the control group, it increased from 1.65 ± 0.21 mm to 2.90 ± 0.20 mm. The difference was statistically significant (P<0.001).
Stähli *et al*. 2020 (^21^)	RCT	40	Miller Class I, II or III single or multiple (RT 1 and 2 Cairo)	MCAT	MCAT + EMD + SCTG	MCAT + STCG	At 6 months, recession depth was reduced from 4.0 ± 1.2 mm to 0.9 ± 1.3 mm in the test group and from 4.5 ± 2.0 mm to 1.0 ± 1.0 mm in the control group. Mean root coverage was 78 ± 26% in the test group and 77 ± 18% in the control group, with no significant differences between the groups.
Zuhr *et al*. 2020 (^22^)	RCT	23	Miller Class I or II, RT 1 Cairo	Tunnel and CAF	Tunnel + CTG	CAF + EMD	At 24 months, digitally evaluated CRC was present in 60.0% of the TUN + CTG group and 0.0% of the CAF + EMD group. RC amounted to 94.0% in the TUN + CTG group and 57.3% in the CAF + EMD group. REC reduction (RECred) was significantly higher for TUN + CTG (1.81 ± 0.56 mm) than for CAF + EMD (0.90 ± 0.45 mm).
Dias *et al*. 2022 (^23^)	RCT	16	RT 1 Cairo	CAF	CAF + SCTG + EMD	CAF + SCTG	The test group showed significantly higher root coverage (86%) compared to the control group (66%) after 6 months. Recession height (RH) and recession width (RW) were significantly reduced in both groups, with better results in the test group. No significant differences were observed in KTH and GT between the groups.
Stähli *et al*. 2023 (^24^)	RCT	24	RT 1 and RT 2 Cairo	MCAT	MCAT + CTG + EMD	MCAT + CTG	At 5 years, 57.14% of the test group and 60% of the control group showed complete root coverage. Mean root coverage (MRC) was 73.87% in the test group and 75.04% in the control group. Keratinised tissue (KT) increased significantly in both groups, from 1.14 mm to 3.07 mm in the test group and from 1.24 mm to 3.02 mm in the control group. No statistically significant differences were found between the groups in terms of CRC, MRC, KT, or RES.
Rieder *et al*. 2024 (^25^)	RCT	15	Miller class I or II, RT 1 Cairo	CAF	CAF + XDM + EMD	CAF + XDM	No statistically significant difference in root coverage between the control group (69 ± 28%) and the test group (36 ± 32%) (p = 0.094). Both groups showed significant improvement in KTW over time (p < 0.001), with no significant difference between groups (p = 0.690). The control group showed superior results in keratinized tissue thickness (KTT) compared to the test group (p = 0.044).
Aydinyurt *et al*. 2019 (^26^)	RCT	19	Miller class I or II, RT 1 Cairo	CAF	CAF + SCTG + Emdogain	CAF + SCTG	Both techniques showed a significant reduction in gingival recession. The EMD group showed better results in soft tissue texture and mucogingival junction alignment (p < 0.05). The complete root coverage rate was 68% in the EMD group and 52% in the control group.

The analyzed studies compared different surgical techniques for the treatment of gingival recessions, focusing on the percentage of root coverage (RC), clinical attachment gain (CAL), increase in gingival thickness (KTW), long-term stability, and postoperative pain (VAS). Clinical measurements performed in the studies were included to allow comparison of results. The analysis of the articles also showed that the Coronally Advanced Flap (CAF) technique is the most commonly used (five studies), often associated with subepithelial connective tissue graft (SCTG), connective tissue graft (CTG), and xenogeneic dermal matrix (XDM). The main results were summarized in Table 3. A total of 228 patients were evaluated in the studies, and a total of 549 were part of the study. The use of Enamel Matrix Derivative (EMD) is associated with these techniques, and its combination with other techniques showed better results in terms of postoperative outcomes and aesthetic perception. The criteria by which these results were achieved include the evaluation of the percentage of root coverage, clinical attachment gain, increase in gingival thickness, long-term stability, and postoperative pain.

## DISCUSSION

This literature review analyzed randomized clinical trials (RCTs) published in the last five years that investigated the use of enamel matrix derivative (EMD) in root coverage procedures for the treatment of gingival recessions. The results of the included studies show a variety of findings regarding the efficacy of EMD in root coverage, clinical attachment gain, reduction of sensitivity, and aesthetic perception.

### EMD and root coverage

The use of EMD has been associated with positive outcomes in terms of root coverage, especially in more complex gingival recessions such as those classified as Cairo RT2 or Miller Class III and IV. Mercado *et al*. ^20^ evaluated the use of EMD in combination with Connective Tissue Graft (CTG) in Miller Class III and IV recessions (Cairo RT2 and RT3) over 36 months. The results showed a significant reduction in recession depth (from 5.71 mm to 1.57 mm in the EMD + CTG group) and stable root coverage over time ^20^. It also resulted in a significant increase in the width of keratinized tissue (from 1.51 mm to 4.18 mm), demonstrating that the use of EMD can improve not only root coverage but also the quality of gingival tissue ^20^. Stähli *et al*. (^24^) followed patients for 5 years after the treatment of Cairo RT1 and RT2 recessions with EMD + CTG ^24^. Complete root coverage (CRC) was achieved in 57.14% of cases in the EMD + CTG group, with an average root coverage (MRC) of 73.87% ^24^. The stability of results over 5 years reinforces the efficacy of EMD ^24^. However, no statistical difference was shown with the control group. 

Stähli *et al.*^21^^)^ did not find significant differences between MCAT + SCTG + EMD and the control group (78% vs. 77% of RC), suggesting that EMD may not be decisive in all techniques ^21^. Górski *et al*. ^18^^)^ showed no significant differences in root coverage between MCAT + SCTG + EMD and the control group (87.4% vs. 90.9%), reinforcing that EMD may not be decisive in all techniques ^18^. Rieder *et al*. ^25^ also observed no advantages of CAF + XDM + EMD (test 36% vs. 69% in the control), indicating that factors such as the xenogenic matrix (XDM) may limit its efficacy ^25^. Aydinyurt *et al.*^26^ reported a higher rate of CRC in the EMD group (68% vs. 52%), highlighting its positive impact on Miller Class I/II or Cairo RT 1 recessions ^26^. Dias *et al*. ^23^ reinforced this finding, showing that CAF + SCTG + EMD achieved 86% complete root coverage, compared to 66% in the control group ^23^.

### EMD and tissue thickness

The use of EMD has shown a positive impact on the thickness of the keratinized tissue (KT) and the quality of the gingival tissue. Mercado *et al*. ^20^ indicates that the group treated with EMD showed a significant increase in the width of the keratinized tissue (KT) from 1.51 mm to 4.18 mm after 36 months, a result significantly greater than in the control group ^20^. Similarly, Stähli *et al*. ^24^ reported an increase in the width of the keratinized tissue (KT) from 1.14 mm to 3.07 mm after 5 years in the group treated with EMD, demonstrating the long-term stability of the treatment ^24^. However, Zuhr *et al*. ^22^ observed that the group treated with TUN + CTG (without EMD) had a significantly greater gingival thickness (GT) (1.41 mm) compared to the group treated with CAF + EMD (0.78 mm), suggesting that CTG may be superior to EMD in terms of increasing gingival thickness ^22^.

Some studies have shown neutral or limited results. Stähli *et al*. ^21^ did not observe significant differences in the width of the keratinized tissue (KT) between the groups treated with and without EMD after six months ^22^. Rieder *et al*. also reported that the control group (without EMD) had superior results in the thickness of the keratinized tissue (KTT) compared to the group treated with EMD ^25^. Górski *et al*. ^18^ did not observe significant differences in gingival thickness (GT) or keratinized tissue width (KTW) between the groups with and without EMD, despite the aesthetic improvement ^18^.

### EMD and inflammatory markers 

Dias *et al*. ^23^ reported that VEGF levels were significantly higher in the test group after 14 days, suggesting increased angiogenesis and healing ^23^. IL-1β and IL-6 levels were significantly elevated in both groups after 7 days, with IL-1β remaining elevated in the test group after 14 days ^23^. Stähli *et al*. ^21^ did not observe significant differences in the levels of inflammatory markers (IL-1β, IL-8, IL-10, MMP-8, and TGF-β1) between the two groups at any time ^21^. Gerova-Vatsova *et al*. ^27^ observed that EMD helps to improve and accelerate the healing of soft tissues and the resolution of inflammation ^27^. Villa *et al*. ^28^ highlighted that TGF-α plays a crucial role in the proliferative phase of wound healing, angiogenic processes, and granulation tissue formation ^28^.

### Aesthetic perception and patient satisfaction

Esthetic perception and patient satisfaction are critical aspects, and EMD stands out in these criteria. Górski *et al*. ^18^ reported less postoperative pain and better esthetic evaluation (RES) in the EMD group, highlighting the alignment of the mucogingival junction and tissue texture ^18^. França-Grohmann et al. observed greater patient satisfaction (evaluated by VAS) and better professional evaluation (QCE) in the EMD group, even without statistical differences in root coverage ^19^. Aydinyurt *et al*. ^26^ highlighted the improvement in tissue texture and mucogingival alignment in the EMD group, key factors for treatment acceptance ^26^. Mercado *et al*. and Stähli *et al*. reinforced that patient treated with EMD reported greater satisfaction, referring to an improvement in associated symptoms such as pain, supporting the idea that the use of EMD generates significant biological benefits and clinical outcomes ^20^^,^^24^.

## LIMITATIONS

The heterogeneity of the included studies, particularly in surgical techniques and graft harvesting methods, may have affected result comparability. Methodological quality also varies, with some studies showing a risk of bias due to lack of blinding or inadequate randomization. The limited number of clinical trials and variability in follow-up periods make conclusions uncertain. However, Mercado *et al*. ^20^. and Zuhr *et al*. ^22^ provide long-term follow-up. Mercado’s study showed good stability in challenging Miller Class III and IV recessions (RT2/RT3 of Cairo), while Zuhr’s study, using Emdogain without grafting, showed inferior outcomes. Graft association has been shown to enhance stability and tissue gain over time ^6^. More long-term randomized clinical trials with standardized methods are needed to clarify the role of enamel matrix derivative in clinical outcomes.

## CONCLUSIONS

The results related to root coverage vary depending on the surgical technique and the type of recession treated. In complex cases such as Cairo RT2 or RT3, it may offer benefits; however, in less complex recessions, the results seem to be more inconsistent. Its capacity to increase gingival thickness may vary depending on the surgical technique and the type of recession. In terms of aesthetics, it appears to be consistently associated with better aesthetic outcomes and greater patient satisfaction, regardless of statistical differences in clinical parameters.
